# The complete mitochondrial genome of the extinct Pleistocene horse (Equus cf. lenensis) from Kotelny Island (New Siberian Islands, Russia) and its phylogenetic assessment

**DOI:** 10.1080/23802359.2019.1699877

**Published:** 2019-12-13

**Authors:** Artem V. Nedoluzhko, Fedor S. Sharko, Eugenia S. Boulygina, Svetlana V. Tsygankova, Natalia V. Slobodova, Natalia M. Gruzdeva, Sergey M. Rastorguev, Natalia N. Spasskaya, Evgeny N. Maschenko

**Affiliations:** a Faculty of Biosciences and Aquaculture, Nord University, Bodø, Norway;; b National Research Centre Kurchatov Institute, Moscow, Russia;; c Research Center of Biotechnology of the Russian Academy of Sciences, Institute of Bioengineering, Moscow, Russia;; d Zoological Museum of Moscow Lomonosow State University, Moscow, Russia;; e Borissiak Paleontological Institute, Russian Academy of Sciences, Moscow, Russia;; f Academy of the Sciences Sakha (Yakutia) Republic, Yakutsk, Russia

**Keywords:** Mitochondrial genome, Pleistocene horse, Equidae, ancient DNA, evolution

## Abstract

The complete mitochondrial genome from the Pleistocene stallion horse (*Equus cf. lenensis*) which complete skull was found in 1901 on Kotelny Island (New Siberian Archipelago, Sakha Republic, Russia) is published in this paper. The mitochondrial DNA (mtDNA) is 16,584 base pairs (bp) in length and contained 13 protein-coding genes, 2 rRNA genes, 22 tRNA genes. The overall base composition of the genome in descending order was 32.3% – A, 28.5% – C, 13.4% – G, 25.8% - T without a significant AT bias of 58.2%.

Lenskaya or Lena horse (*Equus cf. lenensis* Russanov, 1968) is extinct Equidae species, which roamed in the Northeastern Siberia during the Late Pleistocene. Presumably, this species lived up to the Middle Holocene in the Arctic zone of Yakutia (northern of 70° north latitude); the latest remains of a frozen mummy is dated by 5450–5310 cal BP (Boeskorov et al. 2014; 2018).

The investigated Lena horse specimen was collected in 1901 on the south coast of the Kotelny Island (New Siberian Archipelago, Sakha Republic, Russia) by Russian Polar Expedition of 1900–1902 that was led by the Russian Arctic explorer Eduard von Toll (1858–1902). The first description of the Late Pleistocene mammals collection from the Kotelny Island was conducted by M. V. Pavlova (Pavlova 1906). The specimen (part of the cranium in a good safe, that was belonged to a stallion by 5–8 years age old) is stored in the Mammal’s Laboratory of the Borissiak Paleontological Institute, Russian Academy of Sciences (collection number PIN 301/1). The absolute age (AMS) of the sample established by collagen from the lower jaw bone is 21,105 ± 55 cal BP (IGAN/CIR UG6966). Morphological characters of the PIN 301/1 specimen allow to define it as *Equus cf. lenensis*.

Lena horse DNA was extracted from the bone powder at the ancient DNA facilities of the National Research Center “Kurchatov Institute” (Moscow, Russia), following the methodology described previously (Orlando et al. 2013).

DNA-library was prepared using an Ovation® Ultralow Library System V2 (NuGEN, Redwood City, CA). Amplified DNA library was quantified using a high-sensitivity chip on a 2100 Bioanalyser instrument (Agilent Technologies, Palo Alto, CA). To sequence, the DNA-library of the *E. cf. lenensis* specimen the S2 flowcell of Illumina Novaseq6000 genome analyzer (Illumina, San Diego, CA) was used with paired-end reads with 150 base pairs (bp) length.

307,010,901 Illumina paired-end reads were generated for DNA library for PIN 301/1 Pleistocene horse specimen. Obtained Illumina reads were processed through PALEOMIX pipeline (Schubert, Ermini, et al. 2014), mapping was done against the *E. caballus* reference sequence (assembly EquCab3.0) using Bowtie 2 under the “very-sensitive” and “rescale” options. We used mapDamage2 (Jonsson et al. 2013), that implemented in PALEOMIX, to model postmortem DNA damage from nucleotide misincorporation patterns (Figure 1(A)).

**Figure 1.**
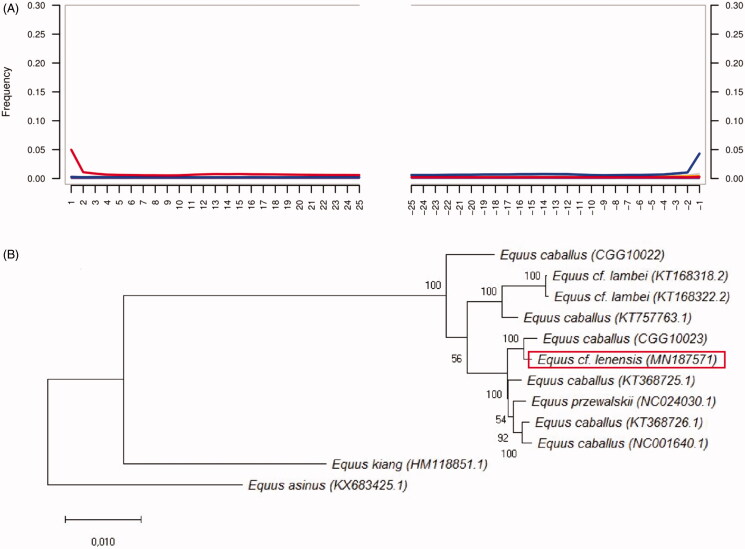
(A) Postmortem DNA damage patterns in DNA reads that were implemented for de novo assembling of Equus cf. lenensis mitochondrial DNA sequence. (B) Maximum likelihood phylogenetic tree reconstruction of the Equidae species, including the extinct Pleistocene Equus cf. lenensis horse based on their CDS.

5,732,968 reads were filtered by PALEOMIX then were used for *de novo* assembling of mitochondrial DNA sequence by a Norgal software package (Al-Nakeeb et al. 2017). The resulting consensus sequence was annotated using the MITOS (Bernt et al. 2013). The obtained annotation was then used to define partitions in the subsequent phylogenetic analysis.

As result, the mitogenome of Pleistocene horse (*E. cf. lenensis*) consists of 16,584 bp (GenBank accession number: MN187571) and includes 13 protein-coding genes (PCGs), 2 rRNA genes and 22 tRNA genes.

Eleven of the 13 PCGs (*NAD6*, *NAD4*, *NAD5*, *NAD4L*, *COB*, *NAD1*, *COX2*, *ATP8*, *ATP6*, *COX3*, *COX1*) used ATG as start codon, another two (*NAD2*, *NAD3*) used ATA. Nine genes (*COX1*, *COX2*, *ATP8*, *ATP6*, *COX3*, *NAD4L*, *NAD4*, *NAD5*, *NAD6*) ended with a TAA stop codon, but for two ones of them (*COX3* and *NAD4*) TAA stop codon is completed by the addition of 3′ A residues to the mRNA, another three (*NAD1*, *NAD2*, *NAD3*) used TAG and *COB* gene ended with a AGA stop codon.

The phylogenetic analysis for coding part of mitochondrial genome sequences (CDS) was performed for the *E. cf. lenensis* (MN187571) and other Equidae species:

- Extinct horse *E. cf. lambei* – KT168318.2, and KT168322.2 (Heintzman et al. 2017);
- Middle Pleistocene horse – KT757763.1 (Orlando et al. 2013);
- Three Late Pleistocene horses – CGG10022, and CGG10023 (Schubert, Jonsson et al. 2014), also as Batagai horse – KT368725.1 (Librado et al. 2015);
- Modern *E. caballus* – KT368726.1 (Librado et al. 2015), and NC_001640.1 (Xu and Arnason 1994);
- Przewalski's horse – *E przewalskii* (NC_024030.1).

The CDS of Tibetan wild ass (*E. kiang*) (HM118851.1), and Qingyang donkey – *E. asinus* (KX683425) were used as an outgroup (Luo et al. 2011; Guo et al. 2017). The phylogenetic relationships were reconstructed using Maximum likelihood analysis was conducted using RAxML. Nodal support was evaluated using 1000 replications of rapid bootstrapping implemented in RAxML (Stamatakis et al. 2008). Maximum likelihood phylogenetic tree reconstruction (Figure 1(B)) has been drown in MEGA X (Kumar et al. 2018).

This study represents the first mitochondrial DNA analysis of *E. cf. lenensis* phylogenetic position based on the available mitochondrial genomes of Equidae species for comparison. Our data show that Lena horse does not represent a separate mitochondrial lineage within *Equus* genus, also as previously published Middle and Late Pleistocene extinct horses from Siberia (Orlando et al. 2013; Schubert, Jonsson et al. 2014; Librado et al. 2015).
